# Involvement of TRPV1 and MOR-NMDAR complex on the antiallodynic effect of LMH-2, a sigma-1 receptor antagonist, in mouse model of diabetic neuropathy - a behavioral approach

**DOI:** 10.1007/s43440-025-00727-4

**Published:** 2025-04-23

**Authors:** Rosa Ventura-Martínez, Guadalupe Esther Ángeles-López, Tania Domínguez-Páez, Gabriel Navarrete-Vázquez, Wendy Arratia-Damián, Maria Eva González-Trujano, Myrna Déciga-Campos

**Affiliations:** 1 Departamento de Farmacología, Facultad de Medicina, Universidad Nacional Autónoma de México (UNAM), Av. Universidad No. 3000, Col. Ciudad Universitaria, Alcaldía Coyoacán, Ciudad de México, C.P. 04510 México; 2 Sección de Estudios de Posgrado e Investigación, Escuela Superior de Medicina, Instituto Politécnico Nacional (IPN), Plan de San Luis y Díaz Mirón s/n. Col. Casco de Santo Tomás, Ciudad de México, 11340 México; 3 Facultad de Farmacia, Universidad Autónoma del Estado de Morelos (UAEM), Cuernavaca, Morelos México; 4 Laboratorio de Neurofarmacología de Productos Naturales, Dirección de Investigaciones en Neurociencias, Instituto Nacional de Psiquiatría “Ramón de la Fuente Muñiz”, Ciudad de México, México

**Keywords:** LMH-2, Antiallodynic effect, σ1R antagonist, TRPV1, MOR-NMDAR complex, Diabetic neuropathy

## Abstract

**Background:**

Recently, the antinociceptive effect of LMH-2, a σ1 receptor antagonist, has been reported in diabetic mice with neuropathic pain. However, the mechanism by which this effect is produced is not completely clear. In this study, we explored the involvement of TRPV1 and the MOR-NMDAR complex in the antiallodynic effect of LMH-2 in hyperglycemic mice with neuropathic pain.

**Methods:**

Hyperglycemia was induced in mice by administering streptozotocin-nicotinamide. Four weeks later, once neuropathic pain was established, the antiallodynic effect of LMH-2 (56.2 mg/kg) was evaluated using the up-down method with the von Frey filaments, both in the absence and the presence of capsazepine (8 mg/kg, *ip*), naloxone (NLX, 1 mg/kg, *ip*), NMDA (0.4 nM/10 µL, *it*), or their co-administration (NLX-NMDA). Gabapentin was used as positive control.

**Results:**

Pretreatment with NLX did not alter the antiallodynic effect of LMH-2 in the up-down method with the von Frey filaments in hyperglycemic mice, whereas NMDA significantly reduced it. The addition of NLX to NMDA (NLX-NMDA) did not modify the effect of NMDA alone on the antiallodynic activity of LMH-2. Additionally, capsazepine completely blocked the antinociceptive effect of LMH-2 in hyperglycemic mice. Molecular docking analysis suggested a potential interaction between LMH-2 and TRPV1. Moreover, a higher dose of LMH-2 did not cause mortality or damage in healthy mice.

**Conclusion:**

These results suggest the potential utility of LMH-2 in the treatment of diabetic neuropathy and highlight a key role for TRPV1 in LMH-2’s antiallodynic mechanism, along with a possible, albeit limited, interaction with the MOR/NMDA complex.

**Supplementary Information:**

The online version contains supplementary material available at 10.1007/s43440-025-00727-4.

## Introduction

Diabetic neuropathy is one of the main complications of diabetes mellitus, where the painful polyneuropathy is produced as a consequence of chronic hyperglycemia [1]. The spontaneous activity and hypersensitivity present in diabetic neuropathy are due to the overactivation of nociceptive C-fibers, which is associated with central sensitization mechanisms and their neurophysiological changes [2–4]. The pathology of diabetic neuropathy is a complex process; therefore, the drugs available for its treatment do not achieve the expected effectiveness, making it necessary to find new therapeutic alternatives.

Recently, the involvement of the sigma-1 receptor (σ1R) in neuropathic pain pathogenesis has been described [5]. The σ1R is a regulatory chaperone protein that modulates some pharmacological targets involved in pain regulation [6]. For this, σ1R antagonists have been proposed as an alternative for pain treatment [7–10]. Haloperidol (HAL), a neuroleptic, induces an antinociceptive effect attributed to its antagonism of σ1R. However, HAL also produces several adverse effects, as agitation, insomnia, depression, and extrapyramidal disorders (associated with movement), which limit its usefulness in neuropathic pain treatment [11–14].

LMH-2 (*N*-(1-benzylpiperidin-4-yl)-4-fluorobenzamide) is a HAL’s analogue with high selectivity for σ1R, which produces antihyperalgesic and antiallodynic effects in models of neuropathic pain [15–16]. LMH-2 shows both a superior analgesic effect and fewer adverse effects than HAL and GBP in hyperglycemic mice, suggesting its potential use as an analgesic for the treatment of neuropathic pain in diabetic patients [16].

Since σ1R is a chaperone protein, LMH-2 may interact with several mechanisms beyond σ1R [16]. In fact, it has been suggested that σ1R antagonism enhances endogenous opioids produced by TRPV1 (transient receptor potential vanilloid 1) neurons, reducing hyperalgesia by increasing µ-opioid receptor (MOR) activity [17]. Additionally, σ1R has been related to a complex formed by MOR, the NMDA ionotropic glutamate receptor (NMDAR), and histidine triad nucleotide-binding protein 1 (HINT1) in neuropathic pain [18]. Within this complex, it has been postulated that under pain conditions, σ1R activation stabilizes NMDARs, promoting their activity. When a σ1R antagonist is administered, it stabilizes the MOR-σ1R complex, increasing MOR activity and decreasing NMDAR activity [18–19]. These events are thought to enhance the analgesic effect of opioid drugs in the presence of a σ1R antagonist. However, the potential involvement of this complex as part of the mechanism of action of σ1R antagonists, such as LMH-2, in the absence of opioid drugs has not been studied.

Furthermore, as the chemical structure of LMH-2 (*N*-(1-benzylpiperidin-4-yl)-4-fluorobenzamide) resembles a fragment of the dimeric opioid peptide p-fluorophenylbiphalin [20] and has been proposed as a heterocyclic peptidomimetic [21], LMH-2 may interact with TRPV1 as part of its antiallodynic effect. To investigate this, we explore the possible involvement of TRPV1 and the MOR-NMDAR complex in the antiallodynic effect of LMH-2 in a mouse model of neuropathy induced by hyperglycemia, as well as the possible interaction of LMH-2 with TRPV1 in silico through docking analysis. Additionally, the acute toxicity of LMH-2 in healthy mice was assessed to contribute to previous safety studies.

## Materials and methods

### Animals

Male CD-1 mice (25–30 g) were obtained from the Bioterium of the Faculty of Medicine of Universidad Nacional Autónoma de México (UNAM). Mice were housed in a controlled environment with a temperature of 22 ± 1 °C and relative humidity of 55 ± 5% under a 12-hour light/dark cycle. The animals were placed in acrylic cages with a removable mesh lid, each cage having a floor area of ​​499.5 cm^2^ (27 cm × 18.5 cm) and a height of 12 cm, housing five mice per cage. All mice were acclimated to the experimental environment for at least five days before testing, with free access to standard food and water. No environmental enrichment was provided. Only sterile sawdust was used for bedding to ensure proper maintenance. Their health status was closely monitored by recording their weight and observing any potential adverse effects following hyperglycemia induction. The experiments were conducted following the Guidelines on Ethical Standards for Investigation of Experimental Pain in Animals [22], the Mexican Official Norm for Animal Care and Handling (NOM-062, 1999) [23], and international guidelines used for Laboratory animals. The protocol was approved by the Institutional Committees of Ethics, Research, and Care and Use of Laboratory Animals of the Faculty of Medicine at UNAM (FM/DI/039/2019). Each animal was used only once and euthanized by cervical dislocation immediately after the experiment.

### Compounds

LMH-2 (*N*-(1-benzylpiperidin-4-yl)-4-fluorobenzamide) was synthesized by Gabriel Navarrete-Vázquez [15]. Citric acid, sodium citrate, nicotinamide (NA), streptozotocin (SZT), naloxone hydrochloride (NLX), N-Methyl-D-aspartic acid (NMDA), and capsazepine (CPZ) were purchased from Sigma-Aldrich (St. Louis, MO, USA).

LMH-2 was dissolved in polyethylene glycol and diluted with saline solution (20%). SZT was prepared in a citrate buffer solution at pH 4.5. CPZ was dissolved in dimethyl sulfoxide (DMSO) and diluted with saline solution (10%) (Sigma Chemical). NA, NLX, and NMDA were prepared with saline solution (0.9% NaCl). All drugs were freshly prepared before administration and given systemically (intraperitoneal or subcutaneously) at a volume of 10 mL/kg, except for NMDA, which was administered intrathecally (*it*) in a 10 µL volume.

### Induction of hyperglycemia in mice

Hyperglycemia was induced on day zero of the experimental protocol by administering a single injection of NA (50 mg/kg, *ip*) following six hours fasting. Thirty minutes later, a single injection of SZT (130 mg/kg, *ip*) was administered (Fig. S1). These single injections of NA-SZT were enough to induce chronic hyperglycemia in mice until day 29, when the experiments were conducted.

Blood glucose concentrations were measured in all mice before and at 3, 7, 14, 21, and 28 days after NA-SZT administration using blood samples obtained by tail vein puncture and analyzed with a commercial glucometer (One Touch Ultra). Only mice with glucose concentration ≥ 250 mg/dL on day seven and that maintained this hyperglycemic state for 28 days were included in the study [16, 24]. A group of normoglycemic animals was included for comparison.

### Evaluation of mechanical allodynia

Mice were placed in acrylic cages with mesh grid floors and allowed to acclimate for 30–60 min before testing. Mechanical allodynia was assessed in normoglycemic and hyperglycemic animals using Von Frey filaments (0.04 to 2 g), and the 50% paw withdrawal threshold was determined for each animal using the up-down method [25]. A positive response to a filament was recorded if the mouse exhibited nociceptive behavior, such as shaking, licking, or abrupt paw withdrawal, upon filament application to the plantar surface of the right paw. The absence of these behaviors was considered a negative response [26]. Each filament was applied perpendicular to the plantar surface and maintained for 2 s at intervals of about 5 to 10 s between each stimulation. The 50% paw withdrawal threshold was determined in all animals before and once a week during 4 weeks after VEH or NA-SZT administration. Mechanical allodynia was considered present when the 50% withdrawal threshold of the paw was less than 0.5 g in hyperglycemic mice; only hyperglycemic animals that developed allodynia were included in this study.

### Experimental design to determine the mechanism of action involved in the antiallodynic effect of LMH-2

All experiments were conducted on day 29, when animals exhibited chronic hyperglycemia and mechanical allodynia. First, the antiallodynic effect of LMH-2 was confirmed in one group of hyperglycemic and allodynic mice at the dose of 56.2 mg/kg by subcutaneous (*sc*) administration, according to previous data from our laboratory. This dose was selected based on a previous study, which demonstrated a dose-dependent effect of LMH-2 (5.6 to 56.2 mg/kg), with 56.2 mg/kg inducing the maximal effect (90%) in this experimental model [16]. Another group of hyperglycemic mice administered with GBP (31.6 mg/kg, *ip*) was used as a positive control. The antiallodynic effect of both LMH-2 and GBP was evaluated every 30 min for 2.5 h (Fig. S1).

To analyze the possible involvement of the MOR-NMDAR complex in the antiallodynic effect of LMH-2, naloxone (NLX, an MOR antagonist, 1.0 mg/kg, *ip*) [27] and NMDA (an NMDAR agonist, 0.4 nM/10 µL, *it*) [28] were co-administered to one group of hyperglycemic animals before LMH-2. Additionally, NLX or NMDA was administered to independent groups of hyperglycemic mice in the absence or presence of LMH-2. NLX was administered 15 min before LMH-2, whereas NMDA was administered five minutes before LMH-2 administration (Fig. S1).

To determine the participation of TRPV1 receptors in the antiallodynic effect of LMH-2, its antagonist, capsazepine (CPZ, 8 mg/kg, *ip*) [29], was administered to another group of hyperglycemic mice, either in the absence or presence of LMH-2. In this case, CPZ was administered 30 min before LMH-2. In all cases, mechanical allodynia was assessed using the Von Frey filaments on day 29 after NA-SZT administration, at 30-minute intervals for 2.5 h following LMH-2 or GBP administration (Fig. S1). To analyze the mechanism of action of LMH-2, we considered the previously proposed mechanism [17, 19, Fig. S2].

### In silico analysis of interaction between TRPV1 receptor and LMH-2 by molecular docking

Docking calculations were performed using the MOE program (Molecular Operating Environment, Chemical Computing Group Inc. Quebec, Canada). The crystal structure of TRPV1 was retrieved from the protein databank with PDB-ID: 5IS0, which corresponds to the co-crystallized structure of the TRPV1 channel with its antagonist CPZ. The Alpha Triangle method was selected as the placement function in MOE, and scores were calculated using the GBVI/WSA scoring function, which estimates the free energy of binding based on force field parameters while accounting for implicit solvation effects. Docking simulations included all residues within a 5.0 Å radius centered on the predefined binding sites of each target. For each ligand, 10,000 conformations were generated before placement. The top 100 placements were subsequently refined using the scoring function. Following molecular docking, the best binding poses were visually inspected, and graphical representations of the ligand-protein interactions were generated for analysis. To validate the method, a redocking of CPZ was conducted with 100 outputs, performed in triplicate, obtaining a score value of -6.73 ± 1.92 and an RMSD of 1.95 ± 0.43 Å. An RMSD (Root Mean Square Deviation) value of less than 2 Å typically indicates that the docking simulation parameters are sufficient to accurately reproduce the ligand’s pose within the protein’s binding site accurately.

### Acute toxicity study

To evaluate the acute toxicity of LMH-2, two groups of healthy mice were used (*n* = 3 per group). A dose three times higher than the maximal antiallodynic dose of LMH-2 in hyperglycemic mice (56.2 mg/kg) was used. This test was conducted following the Organization for Economic Cooperation and Development (OECD) guideline No. 425 [30]. Briefly, one group was treated with a single subcutaneous dose of 175 mg/kg of LMH-2, while the other received the vehicle (VEH). All animals were observed individually every 30 min for 4 h and daily thereafter for a total of 14 days to monitor clinical signs of toxicity or mortality. The body weight of all animals was recorded before treatment administration (day 0) and every 24 h for 14 days.

### Statistical analysis

Animals were randomly assigned to groups of 6 animals per group. Data are presented as the mean ± standard error (SE) of five to six mice per group. The sample size for each group was calculated using the following formula [31]:

$$n=\frac{2\cdot{SD}^{2}\cdot\left(Z_{\alpha/2}+Z_{\beta}\right)^2}{\left({\mu }_{2}-{\mu }_1\right)^2}$$

Where the average values from different experimental groups were 15 (µ_2_-µ_1_), with µ_2_ representing minimal significant relief with clinical relevance (20%) and µ_1_ representing the lack of relief (5%). The standard deviation (SD) was 9, reflecting the expected variability among animals receiving the same treatment. A statistical power of 80% (Z_β_ = 0.84) and a significance level of 95% (α = 0.05, Z_α/2_ = 1.96) were used. Based on these parameters, an *n* = 5.64 was calculated, leading to the use of 5 or 6 animals per experimental group. The total number of mice used in this study was approximately ninety, including those used for acute toxicity.

Blood glucose concentration and the 50% withdrawal threshold were compared between animals with or without NA-SZT administration using two-way analysis of variance (ANOVA) followed by Tukey’s test. Time-course (TC) analyses of the allodynic effect (paw withdrawal threshold in g) in hyperglycemic animals with or without treatment were performed at different points. The area under the curve (AUC) was obtained from the TC using the trapezoid method for each treatment. Statistical differences between treatments were determined by one- or two-way ANOVA, followed by Tukey’s test. A significance level of *p* < 0.05 was considered statistically significant. Statistical analyses were performed using GraphPad Prism version 9.1.1 (GraphPad Software INC, La Jolla, CA, USA).

## Results

### Controls of the model of NA-STZ-induced diabetic mice

**Fig. 1:**
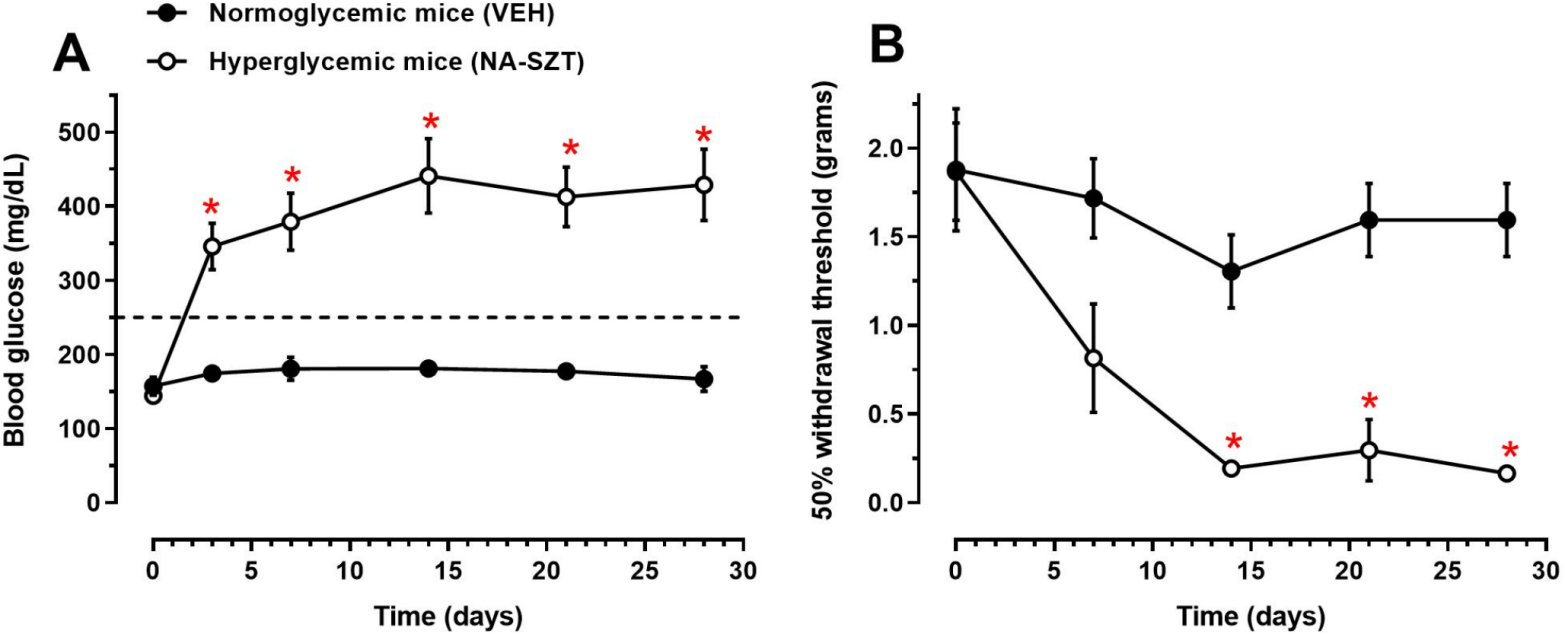
Temporal courses of blood glucose concentration and nociceptive threshold in mice with nicotinamide-streptozotocin (NA-STZ, 5-130 mg/kg, *ip*; hyperglycemic) and with vehicle (VEH, normoglycemic) for 4 weeks in a model of diabetic neuropathy. NA-STZ or VEH were intraperitoneally administered only once on day zero. Blood glucose levels (**A**) and (**B**) mechanical allodynia were measured weekly for 4 weeks post-NA-STZ (hyperglycemic mice) or vehicle (VEH, normoglycemic mice) treatment. Each point represents the mean ± SE of 5 to 6 animals per treatment. *p < 0.05 using a two-way ANOVA followed by Tukey’s test

The development of hyperglycemia and mechanical allodynia in mice following NA-SZT administration was recorded once a week for four weeks. The blood glucose level of the animals four weeks after NA-SZT administration were significantly higher than those recorded before NA-SZT administration (*p* < 0.0001, effect factor F_1,10_=38.57, *p* = 0.0001; time factor F_5,60_=15.6, *p* < 0.0001; interaction F_5,60_=12.06, *p* < 0.0001; subject F_10,50_=6.03; *p* < 0.0001; two-way ANOVA for repeated measurements). Additionally, blood glucose levels in hyperglycemic animals (NA-SZT group) were significantly higher than those in normoglycemic animals on days 3 (*p* = 0.0042), 7 (*p* = 0.0004), 14 (*p* < 0.0001), 21 (*p* < 0.0001), and 28 (*p* < 0.0001) (treatment factor F_1,60_=126.5, *p* < 0.0001; time factor F_5,60_=8.49, *p* < 0.0001; interaction F_5,60_=6.563, *p* < 0.0001; two-way ANOVA followed by Tukey’s test ) (Fig. 1A).

Regarding allodynia development, NA-SZT administration also caused a reduction in the paw withdrawal threshold (g) in hyperglycemic mice starting from week 1 compared to normoglycemic mice (*p* = 0.1428), with significant differences observed at weeks 2 (*p* = 0.028), 3 (*p* = 0.0052), and 4 (*p* = 0.0014) (treatment factor F_1,45_=46.01, *p* < 0.0001; time factor F_4,45_=8.273, *p* < 0.0001; interaction F_4,45_=3.22, *p* = 0.0208; two-way ANOVA followed by Tukey’s test) (Fig. 1B).

### Antiallodynic effect of LMH-2 in hyperglycemic mice

**Fig. 2:**
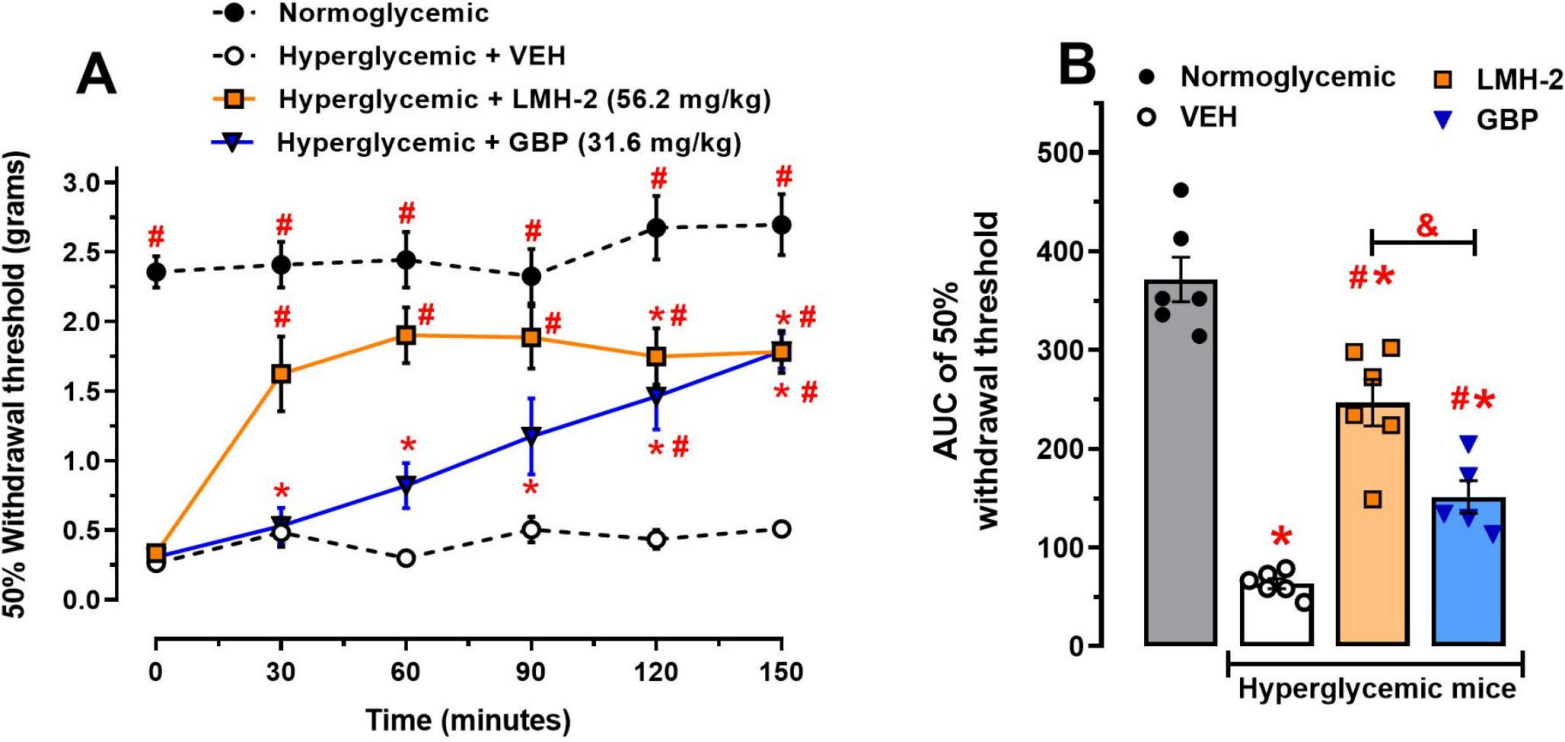
(**A**) Temporal courses (TC) of the nociceptive threshold in hyperglycemic mice with vehicle (VEH), LMH-2 or gabapentin (GBP). Mice were evaluated on the 29th day after administration of nicotinamide-streptozotocin (NA-STZ), once the mechanical allodynia was assessed. The antiallodynic effects of LMH-2 and GBP are represented as the 50% withdrawal threshold (grams) to mechanical stimulus using von Frey filaments. One group of normoglycemic mice was included. Each time course represents the mean ± SE of 5–6 animals per group. # *p* < 0.05 vs. VEH group, * *p* < 0.05 vs. normoglycemic group, two-way ANOVA followed by Tukey’s test. (**B**) Global nociceptive threshold of hyperglycemic mice with VEH, LMH-2 or GBP obtained from their TCs. Each bar represents the mean ± SE of the AUCs obtained from the temporal courses in 5 to 6 animals per group. * *p* < 0.05 vs. normoglycemic group, # *p* < 0.05 vs. VEH group, and ^&^*p* < 0.05 according to one-way ANOVA followed by Tukey’s test

Administration of LMH-2 (56.2 mg/kg) increased the 50% withdrawal threshold in hyperglycemic animals from 30 min after administration compared to VEH (*p* = 0.0006), reaching response levels similar to those of normoglycemic animals (*p* = 0.126). The increase in the 50% withdrawal threshold induced by LMH-2 was considered an antiallodynic effect, which persisted for up to 150 min (*p* < 0.0001). GBP (31.6 mg/kg), used as a positive control, also increased the 50% withdrawal threshold in hyperglycemic animals at 120 and 150 min compared to VEH (*p* = 0.0092 and *p* = 0.0002, respectively). However, GBP did not restore the mechanical threshold to normoglycemic levels as LMH-2 did (treatment factor F_3,114_=171.6, *p* < 0.0001; time factor F_5,114_=13.68, *p* < 0.0001; interaction F_15,114_=4.002, *p* < 0.0001; two-way ANOVA followed by Tukey’s test) (Fig. 2A). In the AUC analyses, LMH-2 showed a superior effect compared to GBP (*p* = 0.0112), representing 72.3 and 44.4% of the antiallodynic effect, respectively (*p* < 0.0001, F_3,19_=51.69, one-way ANOVA followed by Tukey’s test) (Fig. 2B).

### Mechanism of action involved in the antiallodynic effect of LMH-2 in hyperglycemic mice

**Fig. 3:**
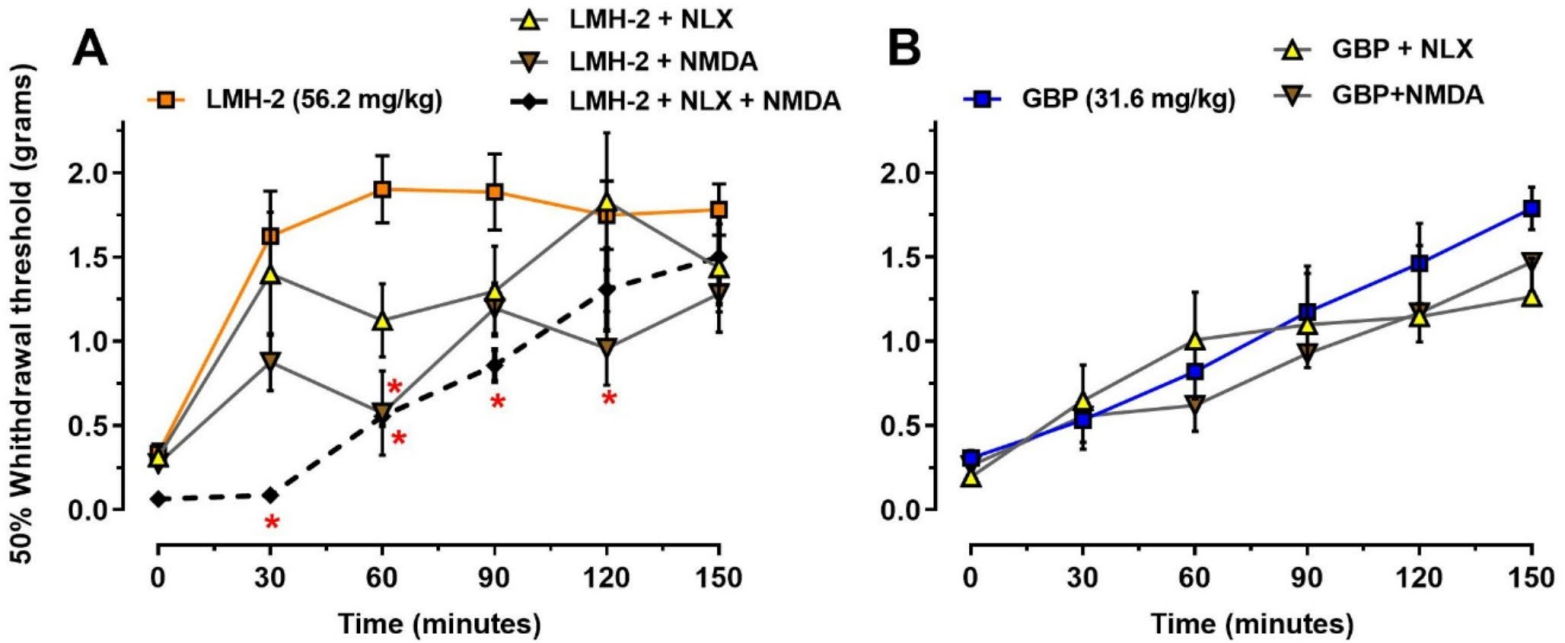
Temporal courses of the antinociceptive effect of LMH-2 (**A**) or gabapentin (GBP) (**B**) in hyperglycemic mice, in the absence or the presence of naloxone (NLX, 1 mg/kg, *ip)*, NMDA (0.4 nM, *it*), or both. NLX was administered 15 min before LMH-2 (56.2 mg/kg) or GBP (31.6 mg/kg), whereas NMDA was administered five minutes before LMH-2 (56.2 mg/kg) or GBP (31.6 mg/kg). Experiments were conducted in hyperglycemic mice on the 29th day after administration of nicotinamide-streptozotocin (NA-STZ), once the mechanical allodynia was assessed. The antiallodynic effect of LMH-2 and GBP were represented as the 50% withdrawal threshold (grams) to mechanical stimulus using von Frey filaments. Each time course represents the mean ± SE of 6 animals per group. * *p* < 0.05 vs. LMH-2 group according to two-way ANOVA followed by Tukey’s test

To investigate the possible involvement of the MOR-NMDAR complex in the antiallodynic effect of LMH-2, the effects of NLX and NMDA alone or in co-administration with LMH-2 were evaluated in hyperglycemic animals. The results showed that NLX slightly reduced the antiallodynic effect of LMH-2 at 60 min (*p* = 0.0528), although the difference was not statistically significant. Conversely, NMDA significantly reduced the antiallodynic effect of LMH-2 at 60 (*p* < 0.0001) and 120 min (*p* = 0.0475). The co-administration of NLX and NMDA significantly reduced the antiallodynic effect of LMH-2 at 30 (*p* < 0.0001), 60 (*p* = 0.0001), and 90 min (*p* = 0.0046) (treatment factor F_3,120_=18.23, *p* < 0.0001; time factor F_5,120_=19.01, *p* < 0.0001; interaction F_15,120_=1.991, *p* = 0.0211; two-way ANOVA followed by Tukey’s test) (Fig. 3A). In contrast, neither NLX nor NMDA altered the antiallodynic effect induced by GBP in hyperglycemic mice (treatment factor F_2,84_=1.071, *p* = 0.3474; time factor F_5,84_=13.93, *p* < 0.0001; interaction F_10,84_=0.49, *p* = 0.8915; two-way ANOVA) (Fig. 3B). NLX or NMDA alone did not alter the mechanical threshold in hyperglycemic mice; whereas NLX + NMDA reduced the mechanical threshold only at 30 min (*p* = 0.0472) (treatment factor F_3,114_ =12.04, *p* < 0.0001; time factor F_5,114_ = 0.2782, *p* = 0.9242; interaction F_15,114_=1.166, *p* = 0.3080) (Fig. S3).

**Fig. 4:**
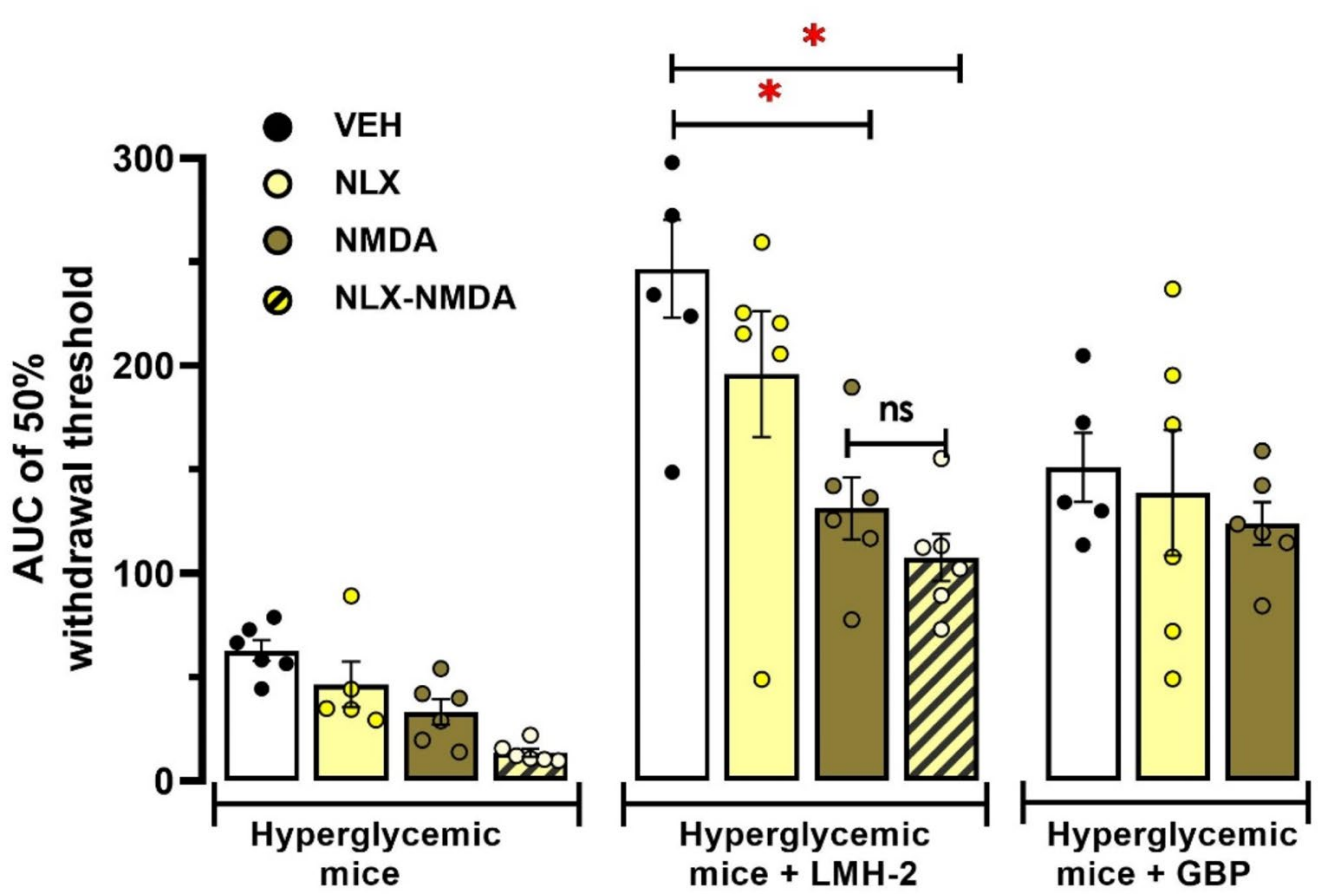
Area under the curves (AUCs) obtained from temporal course of the pretreatment with naloxone (NLX, 1 mg/kg, *ip)*, NMDA (0.4 nM, *it*) or both in the antiallodynic effect induced by LMH-2 or gabapentin (GBP) in hyperglycemic mice. The effect of vehicle (VEH), NLX, NMDA or both compounds in hyperglycemic mice was included. Each bar represents the mean ± SE of the AUC of 5–6 animals per group. * *p* < 0.05 vs. VEH group of the corresponding group of treatments according to two-way ANOVA followed by Tukey’s test

In AUC analysis, NMDA alone and co-administered with NLX significantly reduced the global mechanical threshold in hyperglycemic animals treated with LMH-2 (*p* < 0.0001 in both comparisons). However, in hyperglycemic mice without treatment, neither NLX, NMDA, nor their co-administration significantly reduced the global mechanical threshold compared to the VEH group (*p* = 0.37, *p* = 0.99 and *p* = 0.88; respectively) (treatment factor F_2,58_=51.53, *p* < 0.0001; pretreatment factor F_3,58_=7.62, *p* < 0.0001; interaction F_6,58_=2.42, *p* = 0.037; two-way ANOVA followed by Tukey´s test) (Fig. 4).

**Fig. 5:**
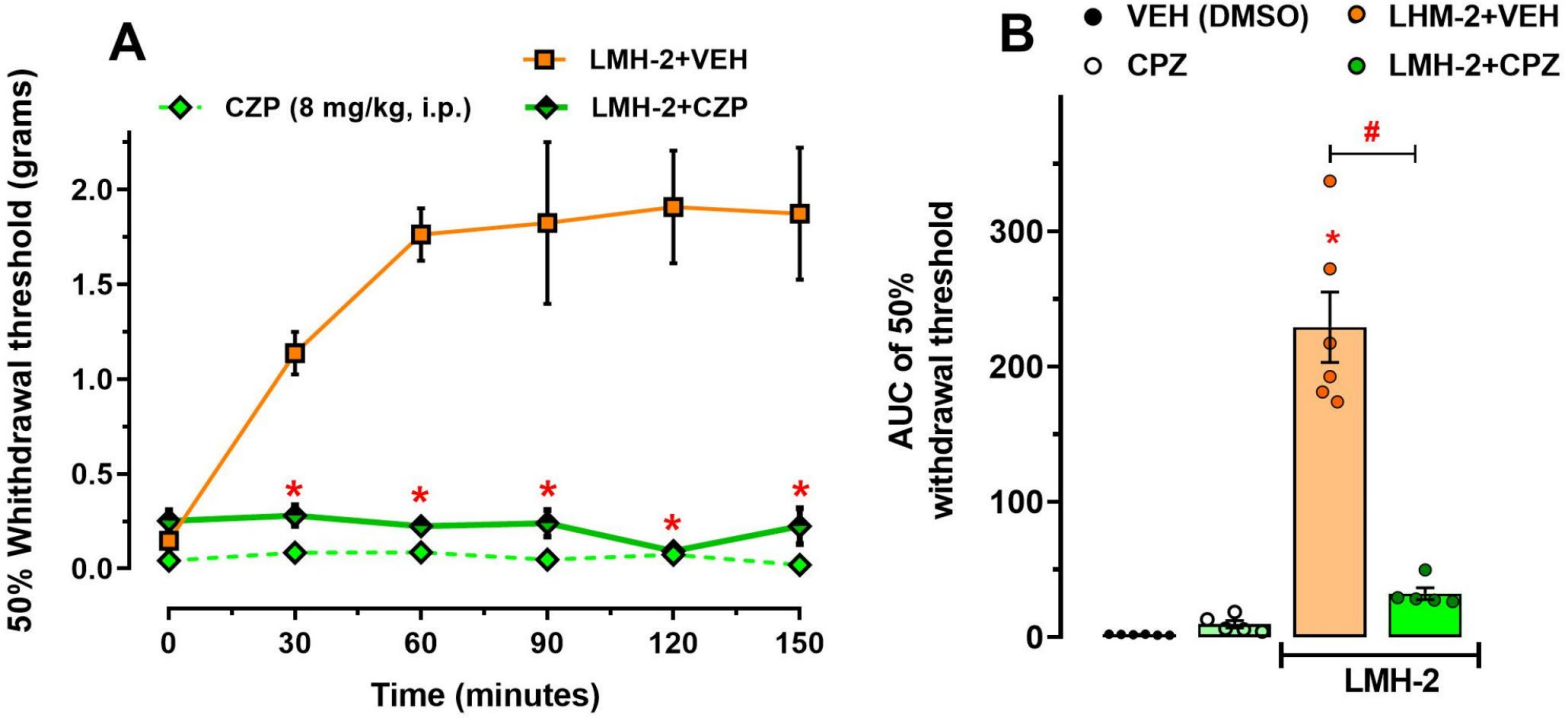
(**A**) Temporal courses of the antinociceptive effect of LMH-2 in the absence or in the presence of capsazepine (CPZ, 8 mg/kg, *ip*), a TRPV1 receptor antagonist in hyperglycemic mice. CPZ was administered 30 min before LMH-2. The effect of CPZ in individual administration in hyperglycemic mice was included as a control. All treatments were evaluated in hyperglycemic mice on the 29th day after administration of nicotinamide-streptozotocin (NA-STZ) once the mechanical allodynia was assessed. The antiallodynic effect of LMH-2 is represented as the 50% withdrawal threshold (grams) to mechanical stimulus using von Frey filaments. Each time course represents the mean ± SE of 5–6 animals per group. * *p* < 0.05 represents the difference between LMH-2 in the absence or presence of CPZ according to two-way ANOVA followed by Tukey’s test. (**B**) Global nociceptive threshold of hyperglycemic mice with vehicle (VEH, DMSO), CPZ, LMH-2, or LMH-2 + CPZ. Each bar represents the mean ± SE of the area under the curves obtained from temporal courses of each treatment in 5–6 animals per group. * *p* < 0.05 vs. VEH, # *p* < 0.05 according to one-way ANOVA followed by Tukey’s test

In another group of hyperglycemic mice, CPZ pretreatment completely blocked the antiallodynic effect of LMH-2 at 30 (*p* = 0.0029), 60 (*p* < 0.0001), 90 (*p* < 0.0001), 120 (*p* < 0.0001), and 150 min (*p* < 0.0001) (treatment factor F_2,78_=112.5, *p* < 0.0001; time factor F_5,78_=4.612, *p* = 0.001; interaction F_10,78_=5.57, *p* < 0.0001; two-way ANOVA followed by Tukey´s test) (Fig. 5A). In the AUC analysis, there was no statistical difference between the effect of CPZ alone in hyperglycemic mice compared to the VEH (DMSO) (*p* = 0.9896); while the LMH-2 effect was almost completely abolished by CPZ pretreatment from 67 to 9.5% of antiallodynic effect (*p* < 0.0001) (F_3,18_= 39.64, *p* < 0.0001, one-way ANOVA followed by Tukey´s test) (Fig. 5B).

### Interaction of LMH-2 and TRPV1 receptor with molecular docking in silico

**Fig. 6:**
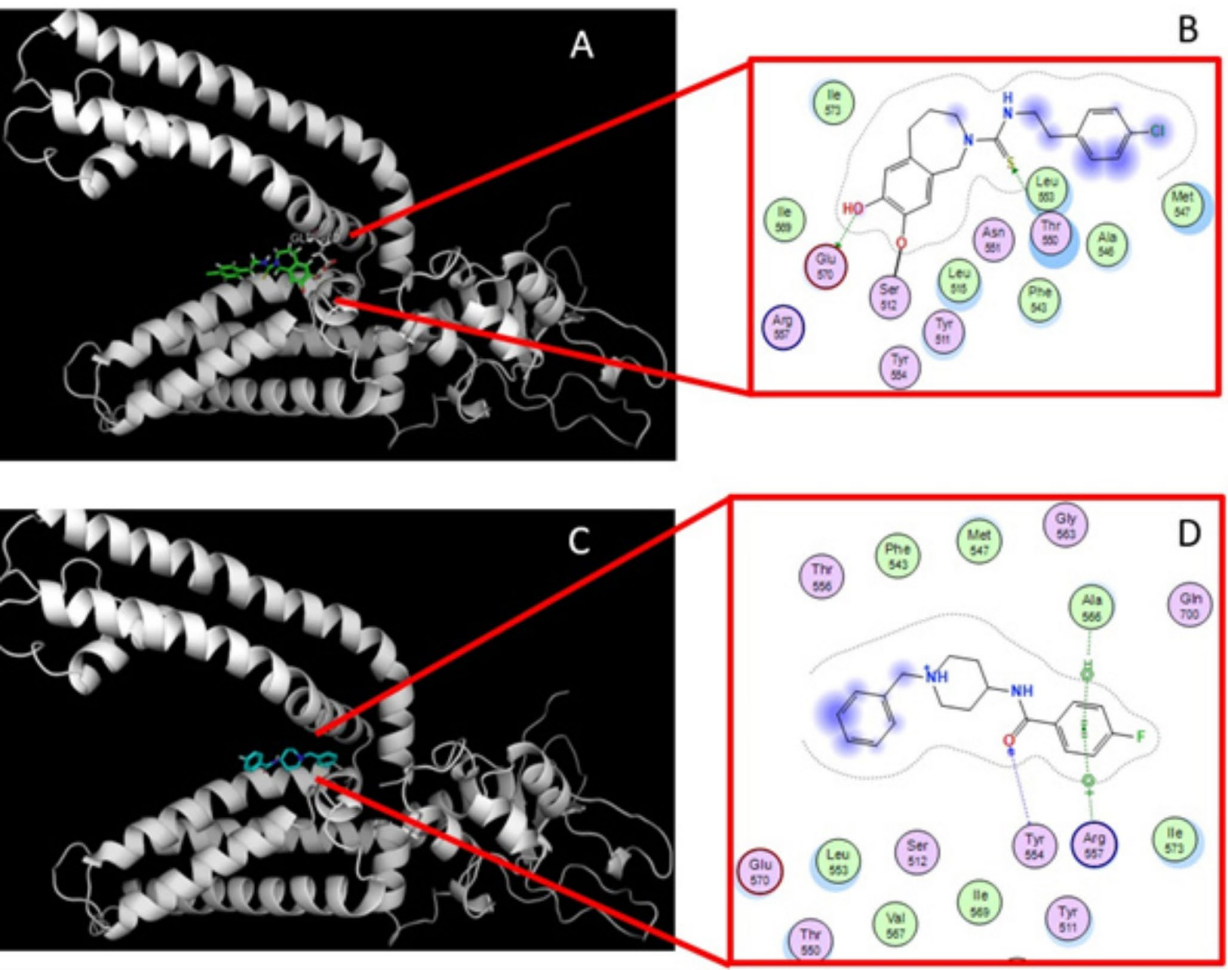
3D and 2D molecular docking models of the TRPV1 ligand-binding site for the antagonist capsazepine (CPZ) (**A**, **B**) and LMH-2 (**C**, **D**) are presented. CPZ (**A**, **B**) occupies the binding site, establishing a series of close interactions with key residues Tyr-554 and Arg-557, which are crucial for TRPV1 channel stability in both closed and open states [32]. Similarly, LMH-2 (**C**, **D**) forms a hydrogen bond with Tyr-554 and a π-cation interaction with Arg-557 within the recognition pocket, contributing to channel stabilization

Figure 6 shows reinforced hydrogen bond interactions between Glu-570 of TRPV1 and the hydroxyl group of CPZ, the co-crystallized TRPV1 antagonist, as well as an additional interaction with Thr-550. Hydrophobic interactions with residues Met-547, Leu-553, Leu-515, and Ile-573 of TRPV1 were also observed. Following validation of the method, molecular docking with LMH-2 yielded a binding score of -6.08 ± 0.12 kcal/mol. The 2D and 3D diagrams reveal that the molecular interactions of LMH-2 with the CPZ-binding pocket into the TRPV1 were conserved, with additional hydrophobic and polar interactions involving Ser-512, Leu-553, Tyr-554, Arg-557, and Ala-566. These findings suggest that LMH-2 may bind to the same recognition site on TRPV1 as the antagonist CPZ [32]. Consequently, the antiallodynic effect of LMH-2 could potentially be blocked by CPZ, as we observed in our experiments.

### Acute toxicity effect of LMH-2

A high dose (175 mg/kg) of LMH-2 did not cause mortality or toxicity signs in mice during the 14-day observation period. No significant differences in body weight were observed between LMH-2 and VEH-treated animals over 14 days (Table 1). No macroscopic changes were detected in internal organs, including the stomach, heart, liver, and kidneys, at 14 days post- administration (data not shown).

**Table 1:** Corporal weight of healthy mice on the acute toxicity test of LMH-2

Treatment	Day								
	Grams	0	1	2	5	8	11	14	
LMH-2 (175 mg/kg, *sc*)	Weight Δ weight	23.3 ± 0.3	25.0 ± 0.0 1.67 ± 0.3	25.7 ± 0.33 2.33 ± 0.3	25.7 ± 0.33 2.33 ± 0.7	26.7 ± 0.33 3.33 ± 0.7	27.7 ± 0.8 4.33 ± 1.2	28.7 ± 0.33 5.33 ± 0.7	
Vehicle (VEH)	Weight Δ weight	20.7 ± 0.9	23.0 ± 1.2 2.33 ± 0.3	24.0 ± 1.2 3.33 ± 0.3	24.3 ± 1.2 3.67 ± 0.3	25.3 ± 1.2 4.67 ± 0.3	25.7 ± 1.3 5.00 ± 0.6	27.7 ± 0.9 7.00 ± 0.0	
	p-value		*p* = 0.99	*p* = 0.98	*p* = 0.89	*p* = 0.89	*p* = 0.99	*p* = 0.64	

Treatment factor F_1,56_=23.09, *p* < 0.0001, time factor F_13,56_=6.63, *p* < 0.0001; interaction F_13,56_=0.14, *p* = 0.99; two-way ANOVA

## Discussion

In the present study, we confirmed that NA + SZT injection induced chronic hyperglycemia in mice from the third day after administration, a condition that was maintained for four weeks. Additionally, chronic hyperglycemia led to the development of allodynia in the animals from the second week, which persisted for four weeks, as previously documented [16]. LMH-2 produced an antiallodynic effect in animals with neuropathic pain induced by hyperglycemia, proving to be more effective than GBP, as previously reported [16]. The antiallodynic effect of LMH-2 was not diminished in the presence of NLX, whereas NMDA significantly reduced it. The addition of NLX to NMDA (NLX-NMDA) did not alter the effects of NMDA alone in the antiallodynic action of LMH-2. On the other hand, capsazepine, a TRPV1 receptor antagonist, completely reversed the antinociceptive effect of LMH-2 in hyperglycemic mice. Molecular docking analysis performed in this study suggested a potential interaction of LMH-2 with TRPV1.

Allodynia induced by chronic hyperglycemia is associated with alterations in motor and sensory nerve conduction velocities in small sensory fibers [33]. There is also evidence that both peptidergic and non-peptidergic nociceptive neurons are selectively damaged by diabetes, contributing to allodynia and hyperalgesia, two hallmark symptoms of neuropathic pain [34]. Moreover, activation of σ1Rs is strongly linked to neuropathic pain, as σ1R antagonists have been shown to alleviate this type of pain [7].

In this context, LMH-2, a novel σ1R antagonist and structural analogue of HAL, has been proposed as an alternative for the treatment of neuropathic pain due to its affinity for σ1R (Ki = 6.0 nM), which is comparable to that of pentazocine (Ki = 5.7 nM) and HAL (Ki = 6.3 nM) [15]. LMH-2 has demonstrated antihyperalgesic and antiallodynic effects in animal models of neuropathic pain induced by sciatic nerve constriction and chronic hyperglycemia, showing greater efficacy than GBP and HAL [15–16]. The antiallodynic effect of LMH-2 reported in our previous study was confirmed in the present work, where a dose of 56.2 mg/kg exhibited superior antiallodynic effects compared to GBP, a clinically used drug for this type of pain.

Since σ1R is a chaperone protein, the antinociceptive effect of its antagonists, such as LMH-2, may involve the regulation of several pharmacological targets, including ionic channels, G-protein-coupled receptors, and neurotransmission systems involved in pain modulation [6]. Recent studies have proposed that σ1Rs modulate the activity of a complex formed by MOR, NMDAR, and HINT1 in neuropathic pain [18]. In this mechanism, σ1R activation enhances NMDAR activity while reducing MOR activity [19, 35]. It has been suggested that σ1R antagonists promote the activity of negative regulators of NMDAR, such as Ca^2+^/calmodulin, thereby decreasing NMDAR signaling and inhibiting pain amplification in the spinal cord. This mechanism is also associated with a reduction in reactive oxygen species production, inhibition of astrocytes and glia activation, and diminished neuronal pain sensitization. Additionally, σ1R antagonists stabilize MOR activity, enhancing its effects [6, 36–38].

It is well established that σ1R antagonists enhance endogenous opioid-induced analgesia (drugs) in nociceptive pain at both central [39] and peripheral levels [5, 19, 40–41]. Part of their antinociceptive effects may involve endogenous opioids naturally produced by immune cells that accumulate at inflamed sites to relieve inflammatory pain [42]. Importantly, the potentiation of opioid-drugs antinociception by σ1R antagonists does not increase opioid-related side effects such as analgesic tolerance, physical dependence, gastrointestinal transit inhibition, or mydriasis. Thus, using antagonists σ1R as opioid-drugs adjuvants could represent a pharmacological strategy to enhance the opioid while increasing their safety margin [43].

However, in the absence of an opioid-drug and based on our results, LMH-2 (a σ1R antagonist) does not appear to interact directly with MOR, as NLX did not alter its antiallodynic effect. This suggests that endogenous opioid production or release does not contribute to LMH-2’s antiallodynic action. In contrast, other studies have shown that during inflammatory injury, the antihyperalgesic effect induced by two selective σ1R antagonists (S1RA and BD-1063) [41, 44–45] was peripherally mediated by endogenous opioids, as their effects were sensitive to NLX [45]. Since neither S1RA nor BD-1063 exhibits affinity for opioid receptors [46–47], their effects were not attributed to direct actions on these receptors.

Regarding the possible involvement of glutamate receptors (NMDAR) in the LMH-2’s antiallodynic effect, our results showed that NMDA administration reduced LMH-2’s efficacy, suggesting that part of its activity may be due to NMDAR modulation. These findings align with a reported study in which S1RA, an σ1R antagonist, regulated pain transmission through its interaction with NMDAR [42, 48]. However, these results are not conclusive, as NMDA administration alone is known to induce pain behaviors [49–50]. Thus, the reduction in the LMH-2 antiallodynic effect in the presence of NMDA may result from NMDAR activation contributing to nociception in hyperglycemic mice rather than a direct role of NMDARs in LMH-2’s mechanism of action. Notably, NMDA administration alone did not increase allodynia in hyperglycemic mice.

To explore the potential involvement of the MOR-NMDAR complex in LMH-2’s antiallodynic effect, we co-administered NLX + NMDA, which diminished LMH-2’s action. However, this effect may be more attributable to NMDA-induced allodynia rather than an interaction between LMH-2 and the MOR-NMDAR complex, as no significant difference was observed between the NMDA + NLX + LMH-2 and NMDA + LMH-2 groups. Additionally, we evaluated the effects of NLX and NMDA on GBP, our positive control. The results showed that neither NLX nor NMDA altered GBP-induced antiallodynic effect, reinforcing the idea that GBP’s effect does not involve endogenous opioids or NMDARs. These findings support the hypothesis that LMH-2 may interact with the MOR-NMDAR complex, consistent with previous studies with other selective σ1R antagonists, such as S1RA and BD-1063 [42].

The main limitation of this study is that the proposed interaction between LMH-2 and receptors such as TRPV1 or NMDAR, as part of its antinociceptive mechanism, are based solely on behavioral experiments. To validate these observations, future research should investigate σ1R’s role in NMDAR modulation using whole-cell patch-clamp recordings in specific regions, such as CA1 pyramidal cells in the mouse hippocampus. Additionally, molecular studies are needed to explore signaling pathways associated with the σ1R-MOR-NMDAR complex, and binding assays should be conducted to determine whether LMH-2 promotes σ1R-MOR complex formation. Electrophysiological studies could further clarify whether LMH-2 modulates NMDAR activity, given that NMDARs are ligand-gated ion channels involved in intracellular Ca²⁺ regulation [49].

On the other hand, σ1R has been suggested to regulate TRPV1, another molecular target involved in pain [17]. In our study, the TRPV1 antagonist (CPZ) completely blocks LMH-2’s antiallodynic effect, suggesting an interaction between LMH-2 and TRPV1. These findings align with previous research demonstrating that σ1R antagonists modulate TRPV1-MOR cross-talk [17]. TRPV1 is a channel involved in the regulation of calcium signaling, which is crucial for many cellular processes [51] (Fig. S2). Also, in this work, we confirmed the interactions between LMH-2 and TRPV1 at the CPZ binding site by the molecular docking *in silico* [32], which supports our experimental findings, although further pharmacological evidence is required to prove this assertion.

Finally, to further assess LMH-2’s safety, we conducted acute toxicity studies following OECD guidelines [30]. An LMH-2 high dose (175 mg/kg) did not induce mortality or toxicity in mice over 14 days. No changes in body weight or macroscopic organ alterations were observed. Additional toxicity studies with higher doses are needed to determine LMH-2’s lethal dose. These results contribute to the safety studies of this σ1R antagonist, LMH-2, reinforcing its potential usefulness in the treatment of pain in diabetic neuropathy.

In conclusion, our findings suggest a key role for TRPV1 in LMH-2’s antiallodynic mechanism and a possible, albeit limited, interaction with the MOR/NMDA complex. Additionally, our results suggested that the endogenous opioid system is not involved in the antiallodynic effect of LMH-2 in this model of diabetic neuropathy. Although molecular and/or electrophysiological studies are required to confirm these mechanisms, these results suggest the potential and sure use of LMH-2 as a treatment for neuropathic pain in diabetic patients.

## Electronic supplementary material

Below is the link to the electronic supplementary material.

Supplementary Material 1

Supplementary Material 2

## Data Availability

The datasets generated during and/or analyzed during the current study are available from the corresponding author upon reasonable request.

## Abbreviations

ANOVA: Analysis of Variance
AUC: Area under the curve
Au: Area units
CPZ: Capsazepine
ER: Endoplasmic reticulum
GBP: Gabapentin
HAL: Haloperidol
HINT1: Histidine triad nucleotide binding protein 1
Ki: Inhibition constant
*ip*: Intraperitoneal
*it*: Intratecal
MOR: Mu-opioid receptor
NLX: Naloxone
NA-STZ: Nicotinamide with streptozotocin
NMDAR: NMDA ionotropic glutamate receptor
NMDA: N-Methyl-D-aspartic acid
σ1R: Sigma-1 receptors
SE: Standard error
*sc*: Subcutaneous
TCs: Temporal courses
TRPV1: Transient receptor potential vanilloid 1
VEH: Vehicle
