# Supplementary figures and images for: Involvement of TRPV1 and MOR-NMDAR complex on the antiallodynic effect of LMH-2, a sigma-1 receptor antagonist, in mouse model of diabetic neuropathy - a behavioral approach
Source: Pharmacol Rep. 2025 Apr 23;77(4):1011–23. doi: 10.1007/s43440-025-00727-4 (PMC12241247; doi:10.1007/s43440-025-00727-4)

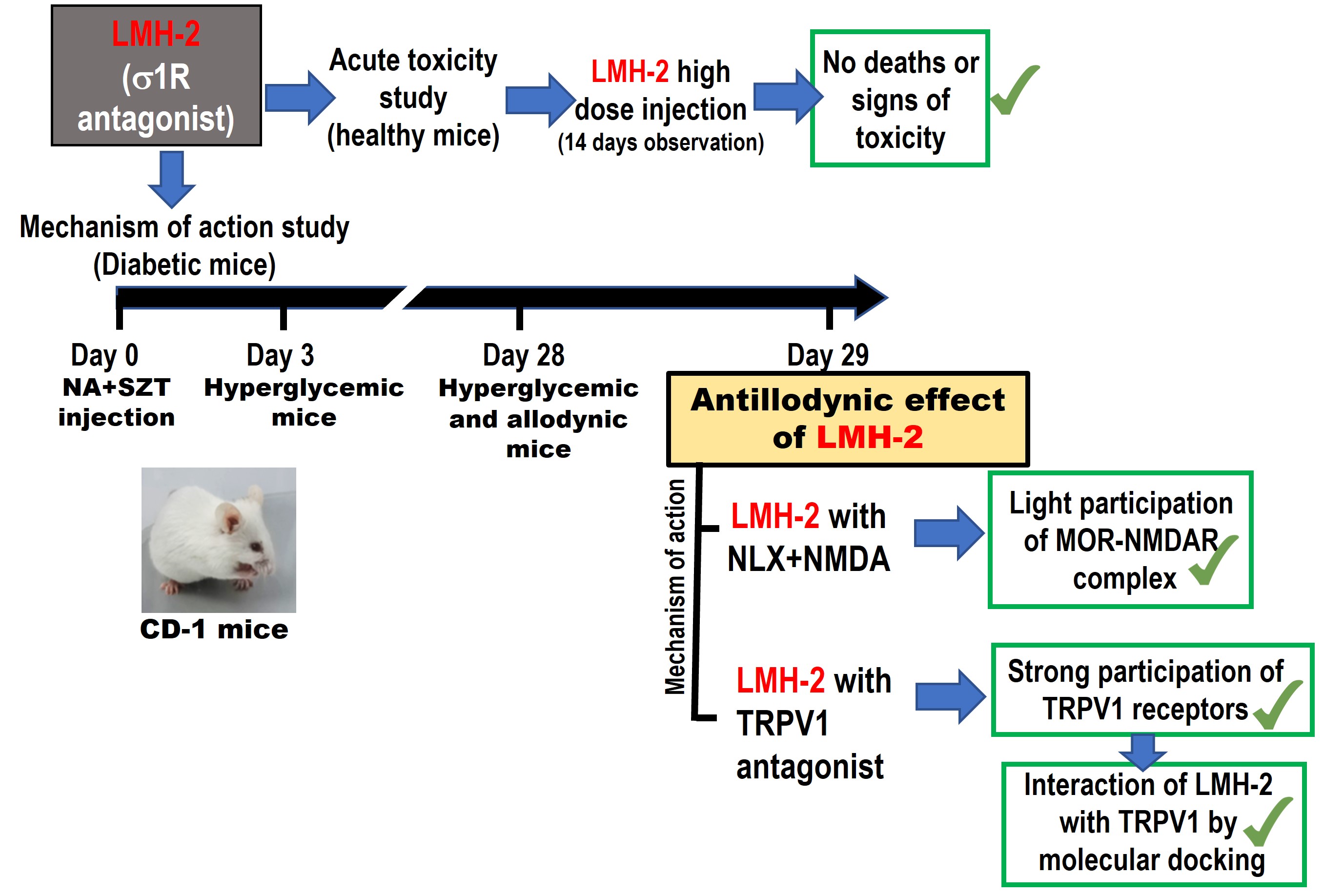

Supplement: Supplementary file 1 — Supplementary Material 1 [file 43440_2025_727_MOESM1_ESM.jpg]
