# Supplementary material for: Involvement of TRPV1 and MOR-NMDAR complex on the antiallodynic effect of LMH-2, a sigma-1 receptor antagonist, in mouse model of diabetic neuropathy - a behavioral approach
Source: Pharmacol Rep. 2025 Apr 23;77(4):1011–23. doi: 10.1007/s43440-025-00727-4 (PMC12241247; doi:10.1007/s43440-025-00727-4)
Supplement: Supplementary file 2 — Supplementary Material 2 [file 43440_2025_727_MOESM2_ESM.docx]

*Supplementary figures for:*

**Title:** Involvement of TRPV1 and MOR-NMDAR complex on the antiallodynic effect of LMH-2, a sigma-1 receptor antagonist, in mouse model of diabetic neuropathy - a behavioral approach

**Journal**: Pharmacological Reports

**Authors**: Rosa Ventura-Martínez, Guadalupe Esther Ángeles-López, Tania Domínguez-Páez, Gabriel Navarrete-Vázquez, Wendy Arratia-Damián, Ma. Eva González-Trujano and Myrna Déciga-Campos

Corresponding authors: 50:50 Rosa Ventura-Martínez and Myrna Déciga-Campos.

**Rosa Ventura-Martínez**, Departamento de Farmacología, Facultad de Medicina, Universidad Nacional Autónoma de México (UNAM). Av. Universidad No. 3000, Col. Ciudad Universitaria, Alcaldía Coyoacán, C.P. 04510. Ciudad de México, México. Phone +52 55 5623 2162. Email: [rventuram@comunidad.unam.mx](mailto:rventuram@comunidad.unam.mx); [rventuram7@hotmail.com](mailto:rventuram7@hotmail.com)

**Myrna Déciga-Campos**, Sección de Estudios de Posgrado e Investigación, Escuela Superior de Medicina, Instituto Politécnico Nacional (IPN). Plan de San Luis y Díaz Mirón s/n. Col. Casco de Santo Tomás,11340. Ciudad de México, México. E-mail: [mdeciga@ipn.mx](mailto:mdeciga@ipn.mx)


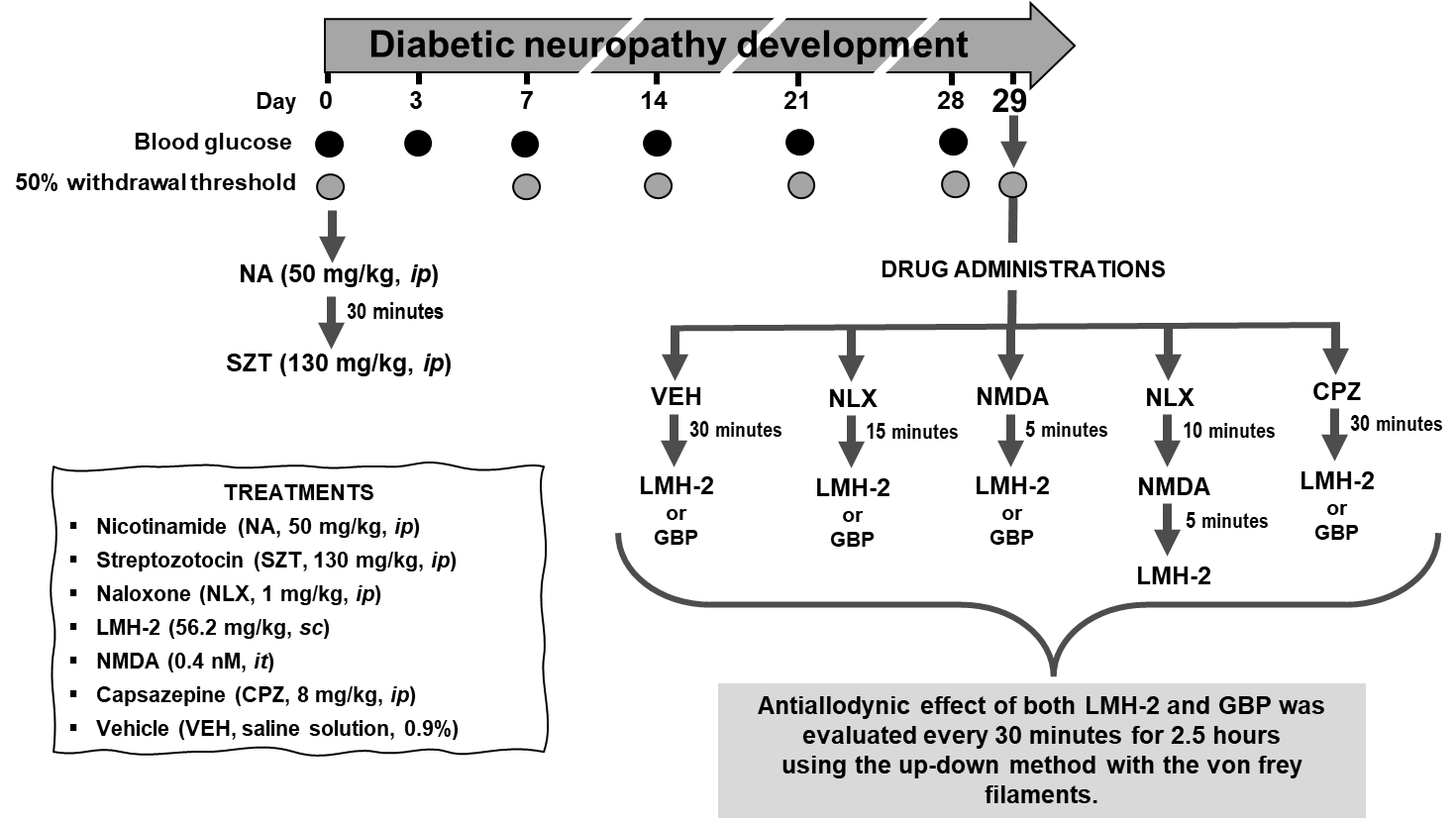


**Figure S1**. Scheme of the experimental design, illustrating drug administrations and tests performed over time. On day zero, hyperglycemia was induced in mice by administering streptozotocin-nicotinamide (SZT+NA). Twenty-eight days later, mice developed mechanical allodynia, which was determined using the von Frey filaments. Experiments (drug administrations) were conducted on day 29. Several treatments were administered to analyze the possible involvement of the MOR-NMDAR complex or TRPV1 receptor in the antiallodynic effect of LMH-2. Gabapentin (GBP) was used as positive control.

**Figure S2.** Proposed mechanism of action for the effects of LMH-2 on allodynia induced by sensitization of TRPV1+ neurons. (A) Sensitization by mechanical stimulus in hyperglycemic mice, favors Ca^2+^ influx through TRPV1. In response to Ca^2+^, the sigma-1 receptor (σ1R) binds to TRPV1, preventing calmodulin (CaM) binding (and therefore preventing desensitization of the channel). TRPV1+ neurons probably produce the endogenous opioid peptide endomorphin-2 (END2), whose effects are insufficient to relieve hyperalgesia. (B) LMH-2 transfer σ1R from TRPV1 to μ opioid receptors (μ), and this facilitates the interaction of CaM with the desensitization site of TRPV1 and the enhancement of the effects of endorphins (opioid agonist), producing opioid-mediated antihyperalgesic effects during nociceptor sensitization [17, 19].

**Figure S3.** Temporal courses of the nociceptive threshold of hyperglycemic mice with vehicle (VEH) in absence or in the presence of naloxone (NLX, 1 mg/kg, *ip*), an opioid antagonist; NMDA (0.4 nM, *it*), an agonist of NMDAR; or both (NLX +NMDA). NLX and NMDA were administered 15 and 5 minutes before the VEH, respectively. Mice were evaluated on the 29th day after administration of nicotinamide-streptozotocin (NA-STZ) once the mechanical allodynia was assessed. The 50% withdrawal threshold (grams) to mechanical stimulus was determined using von Frey filaments. Each time course represents the mean±SE of 5-6 animals per group. * p<0.05 vs VEH group. Two-way ANOVA followed by Tukey's test.
